# Surgery

**Published:** 1893-09

**Authors:** John Parmenter


### SURGERY.

Conducted by JOHN PARMENTER, M. D.

Dr. Lanphear, (*American Journal of Surgery and Gynecology*, June, 1893,) after describing briefly the methods of Senn and Wyeth, giving the preference to Wyeth's procedure, goes on to relate four cases of bloodless amputation of the hip, operated upon by his own modification of the Wyeth method. This consists in inserting the outer pin higher up and drawing the rubber tube very tightly, and has the advantage of completely preventing hemorrhage and permitting disarticulation of the femur without preliminary amputation, thus saving considerable time. Of the four cases, three recovered and one died subsequently from septicemia, the very condition for which the amputation was made. The operation in this particular case was completed in twenty-nine minutes from the beginning of anesthesia. The author concludes with the following remarks :

So far as I can learn, twelve cases have been operated on by this method, with two deaths—a mortality rate of less than seventeen per cent. The significance of this will be appreciated when we recall the facts that heretofore hip-joint amputation has been regarded as one of the most fatal and bloody operations ; that the death-rate has been from sixty per cent. to ninety per cent. by other methods ; and that seventy per cent. of all deaths have been from hemorrhage ! Here are *twelve cases without any hemorrhage*—with one death from shock and one from septicemia. Surely this is a method which is about as successful as could well be expected, considering the gravity of the operation. It certainly controls bleeding completely—the great desideratum, since it is hemorrhage that usually causes death, that brings on "shock," (in most instances) that predisposes to the development of germs introduced during the operation, and, finally, that retards the process of healing.

---

KEEN (*American Journal of Surgery and Gynecology*, June, 1893,) describes a case of apparent intestinal paralysis, which caused arrest of the intestinal contents, and was equivalent to intestinal obstruction, and for which he performed laparatomy. The history is so minutely, yet simply, given, the treatment so scientific, and the deductions so clear and logical, that we reproduce the article *in toto* :

Mrs. S., *æt.* forty-five, mother of several children, has had a great deal of sorrow and consequent mental distress for the last few months. Was first seen by me in consultation with Dr. S. Mason McCollin on the evening of December 24, 1890.

Some months ago, Dr. McCollin removed a soft polyp from the mouth of the womb, and the patient returned to him from time to time for treatment after its removal. Of late, she has had a very offensive vaginal discharge, so much so that Dr. McCollin suspected possible cancer of the uterus, and it was so diagnosticated by another competent gynecologist, who had even advised the removal of the entire uterus the day before I saw her.

On December 18, 1890, the patient came to town, and reached Dr. McCollin's office utterly exhausted. He put her to bed and gave her some vaginal douches to relieve the odor. She seemed somewhat dazed on the 18th and 19th ; on the 20th, she became delirious, and her strength failed to such an extent that Dr. McCollin feared she would die during the night. From then until the 24th she has been in a precarious condition. There has been much elevation of temperature, and she has been continually out of her head, and has had an excessively fetid breath. On the 23d and 24th she vomited a great deal, sometimes for nearly an hour at a time. She said she had had a movement of the bowels on the 19th, but this was not confirmed. From that time until now there has been no movement whatever, nor has any flatus escaped, and yesterday (23d) an enema of half a gallon was entirely retained. No gurgling in the bowels has been heard. Her menstruation began today.

December 24, 1890. On examining the abdomen no especial tenderness was discovered, excepting over the right iliac fossa, and not very much there. There was also, it seems to me, more resistance at that point. On examining the bowel, I found that the rectum was absolutely empty. It was "ballooned" into a cavity, certainly as large as the fist. No strictures were observed, and the tube with which the half-gallon enema had been injected had been inserted eighteen inches into the bowel, with no obstruction. On examining the uterus, it was found to be movable, not enlarged, the os slightly patulous, so that the tip of my forefinger could be inserted for a scant half inch, and felt the stump of the polyp. There was no cauliflower or other growth about the womb. The odor of the menstrual discharge was not offensive.

Inasmuch as she seemed to be going down hill very rapidly, and as she would certainly die very shortly if nothing was done, I recommended an exploratory laparatomy on the next day, unless a large enema, with elevation of the hips, should be followed by any result.

December 25, 1890. Dr. McCollin reports that almost a gallon of fluid was injected last night and the hips were elevated. The entire enema was retained. She was even more delirious last night than before.

*Operation.*—An incision was made in the median line, which was eventually extended to a little above the umbilicus. As it was found that there was a marked tendency towards an umbilical hernia, the entire umbilicus was dissected out and removed. There were no adhesions, no fixation of the pelvic viscera, no intussusception, (which to my mind, in spite of her age, was the most likely prior diagnosis,) and no band or other obstruction to be found. The large intestine, as had been determined by percussion before the operation, was enormously distended, and the small intestine, in its lower half, was contracted and empty, but in its upper half evidently contained feces. The large bowel had a rather sharp V-shaped attachment to the spleen, but not enough to produce any obstruction. At two points in the ascending colon and the sigmoid flexure an incision, over half an inch long, was made in the large bowel, and an enormous quantity of gas let out. The intestinal incisions were then closed with Cushing's right-angled continuous suture. The large bowel was followed from the rectum to the cecum, and the small bowel rapidly gone over from the cecum to the duodenum, but no obstruction was found. The gall-bladder was distended with bile. Both it and the liver were healthy. The abdomen was then closed as speedily as possible. A glass drainage-tube was inserted, going down into Douglas' cul-de-sac. Before the patient had been removed from the table, the bowels were freely moved. During the operation she suffered deeply from shock. This was admirably relieved by hypodermatics of strychnine. The urine had been examined repeatedly, and found normal, but urine drawn a few minutes before the operation, and examined afterwards, showed moderate albuminuria.

December 26, 1890. She passed a very bad night, being almost wildly delirious at times, so that she had to be restrained by physical force. She has made no complaint of pain, but if the abdomen is pressed upon she winces as though hurt. Just after the operation the drainage-tube was cleaned by suction every fifteen or twenty minutes, blood-stained serum being removed. The intervals were rather rapidly lengthened, until this morning, after five hours, only one drachm had accumulated. This fluid is entirely sweet. Her temperature is only a little above 100; tongue coated; breath still very foul. Her pulse has increased in frequency to 124 and has lost in force. Since the operation absolutely nothing has been given her, except a little ice and champagne, and a little brandy and water. In view of the paralysis which had existed, at least in the large bowel, and possibly in the lower part of the small bowel, I deemed it best for her simply to be stimulated moderately and have no food whatever for at least twenty-four hours. At 11 A. M. to-day, a distinct gurgling was heard in the intestine, and has continued at intervals since. A rectal tube was inserted once in four hours, and has given exit to some gas, and she has also passed some spontaneously, the first in seven days.

The drainage-tube was removed after twenty-eight hours, there being scarcely any further accumulation of fluid in it. Her general condition was very bad, and it looked as if she could live only a few hours. The wound itself looked perfectly well. During the afternoon she rapidly failed, and for a short time there was no appreciable pulse even at the wrist, while her respirations were shallow, quick, and irregular. Once or twice it seemed that the respirations were permanently suspended. There again, *strychnine*, hypodermatically administered, was of the greatest value.

December 29, 1890. On the 27th (second day), to my surprise, she was somewhat better, and we began cautiously to feed her a little milk and liquid peptonoids only. Flatus occasionally escaped spontaneously, and almost always whenever the tube was inserted, and not uncommonly small quantities of fecal matter also were passed by the tube. On the 28th (third day) she was about the same physically, but her mental condition had improved. The wound, on examination, appeared to be in perfectly good condition. Two or three doses of sulphonal, in ten-grain powders, were enough to reduce and almost to control her restlessness.

January 1, 1891. By the 30th (the fifth day), her temperature had touched the normal, with a slight rise at night. On the 31st (the sixth day), as her bowels had not been freely opened, we decided to give her a dose of thirty grains of Epsom salt every two hours, very cautiously to empty the bowels. Soon after she had the second dose, and not probably as a result of it, but accidentally, an attack of diarrhea began, and within twenty-four hours she had thirty to forty liquid movements, a number of them of considerable amount. The odor, especially of the earlier ones, was excessively fetid. With this diarrhea her mind seemed to clear up considerably, so that she herself observed the odor of the passages; but she developed a great disinclination to take either food or medicine, due chiefly, it seemed, to her mental condition. The wound had healed throughout by first intention, and I removed today all the superficial and half the deep sutures. Her temperature was normal.

January 3, 1891 (ninth day). The remaining sutures were removed today. Her mental condition is very much improved. The bowels are a great deal better and have lost their excessively offensive odor, as a result, probably, of the administration first of salol and then of naphthaline, especially the latter. Two thirty-grain doses of bismuth, following several drachm doses of sulphate of magnesia, seem to have arrested the diarrhea completely.

From this time on her recovery was progressive, but slow. Her mental condition especially was clouded for a considerable time, but eventually she recovered entirely.

June 12, 1891 (six months). She saw me today, and was in better health than she has been for years past. Her mental condition is entirely restored.

*Remarks.*—There are several interesting features in this case which demand attention. First, the pathological condition. From the middle of the small bowel down to the ilio-cecal valve, the lumen of the bowel was diminished to less than one-half the caliber of the upper portion, whereas the large bowel was dilated very greatly with gas. Neither the constricted half of the small bowel nor the dilated large bowel contained any fecal matter, only a few drops exuding through the two incisions made in the large bowel, and so far as could be judged by stripping with the finger, the contracted small bowel was equally empty. It did not, however, seem to be in a condition of spasm as far as could be judged; there was no rigidity or special hardness. There was no occlusion at the point of sudden narrowing, nor any other extensive constriction, but certain it was that for a week, and possibly longer, no peristaltic gurgling had been heard, nor, from the absence of any fecal matter, had any peristalsis probably existed. My opinion, prior to operation, was that most likely, in spite of the patient's age, there was invagination at the ileo-cecal valve, or, possibly, appendicitis. Her mental condition was such, however, that all the subjective signs were doubtful, and even the objective signs, such as tenderness, etc., were very difficult to establish. I have never before seen apparent arrest by so sudden a contraction of the small bowel and the dilatation of the large bowel without assignable cause.

Whether there was any sapremia as a result of the continued presence of fecal matter in the bowel and absorption of the ptomaines of decomposition, will always be doubtful, but it seems reasonable to suppose that this was the cause of the mental and, to a large extent, of the physical condition. The evidence of this rests on the horribly fetid discharges which took place soon after the operation, with the simultaneous clearing of a very foul tongue, the disappearance of a very fetid breath, and the betterment of her mental condition.

Secondly, the laparatomy was entirely exploratory, and the case shows the wisdom of it. The laparatomy was done to make a diagnosis, as well as to institute such treatment as the conditions found would warrant. The case shows also the wisdom of small incisions in the bowel, incisions which can be perfectly well made and closed by any of the ordinary intestinal sutures, rather than making punctures and leaving them to heal without sutures. The distended large bowel was relieved of its tension and resumed its function, whether by the stimulus of the handling and washing out of the belly-cavity with hot water, or the reëstablishment of the peristalsis of small intestines, I cannot say. Probably each one of these factors had some influence in bringing about these results.

In closing the intestinal incision I used Cushing's continuous right-angled suture, as shown by him in the Boston City Hospital Reports for 1889. I think it very easy of application and very speedy and satisfactory. I did not use the rather complicated method he described of securing the two ends. The first end I

simply tied in an ordinary knot and the last end I slipped through its own loop two or three times and cut it off short.

---

JOSEPH PRICE, (*Virginia Medical Monthly*, August, 1893,) in a lecture before the Post-Graduate School, of Chicago, discusses the Post-operative Sequelæ of Pelvic and Abdominal Surgery. He divides his subject into three headings, viz.:

1. Post-operative Sequelæ Due to Complications Induced by Delay in Operating.
2. Complications Induced by Faulty Work and Methods.
3. Sequelæ which may be said Naturally to Follow any Serious Surgical Procedure of the Nature Under Consideration.

The writer regards delay in operating to be at once "the bane and danger of all pelvic and abdominal surgery—baneful to the surgeon and dangerous to the patient." Pus is to be invariably removed as soon as detected. Equally soon should all tumors of the uterus be extirpated. These conditions not only become worse themselves with time, but induce other and dangerous diseases, such as inflammation in adjacent organs, and adhesions, which latter may only precede gangrene of the bowel. In such cases, intestinal surgery must be viewed from two standpoints, viz.: "Both in the light of operative complications, and of being indirectly the cause of post-operative complications and sequelæ. Its presence as a complication of the original operation for the removal of the pus-tube is incident to delay in the original operation, and is a necessity on account of this delay. Without it, the operation, as at present necessitated, would be a failure or a very bleak success." In such cases, after-trouble is unavoidable, (such as stenosis following gangrene, adhesions, hernia, fistula, etc.,) and must not be laid at the door of the operation, but to the delay which made conditions over which surgery could not completely triumph.

The same reason applies to uterine tumors which have been allowed to grow and adhere to important organs to such an extent as to make their extirpation difficult and dangerous and some of the sequelæ of an almost irremediable character.

The writer's words upon the complications due to faulty work and methods should be read and re-read by every youthful and many experienced operators in pelvic surgery. These sequelæ are many. Here are some of them:

1. Adhesions due to irritant irrigating fluids.

2. Adhesions due to imperfect handling.

3. Tears in the omentum neglected and causing subsequent intestinal strangulation.

4. Unnecessary destruction of the peritoneum.

5. Imperfect breaking up of adhesions between not only parts removed and those which remain, but also those between all remaining parts otherwise healthy. (Failure to do this is a fertile cause of bowel obstructions, resulting fatally in cases that would otherwise recover.)

6. Faulty handling of hemorrhage. (The writer recommends the use of silk rather than catgut, torsion of smaller arteries, avoiding the Trendelenburg position, which often leads to the overlooking of oozing and indirect leakage.)

7. Improper drainage. Dr. Price speaks in no uncertain tone regarding the use of drainage. He believes in it thoroughly, but in its simplest form, viz. : a small glass drainage-tube, reinforced with a long-nozzled syringe.

8. The use of big and unnecessary ligatures, which act as foreign bodies, and cause fistula.

9. The leaving behind of removable diseased organs or conditions, such as vaginal puncture for pus in the tubes and vaginal removal of diseased organs.

10. Ventral hernia, from too early rising, too early laying aside of the bandage, or foolish physical exertion, such as dancing, riding, etc.

The writer concludes his admirable address by referring to some after-conditions of operations which may naturally be expected. He warns his hearers that the phenomena attending removal of the appendages varies in individual cases. Some enjoy immunity from discomfort, others suffer with the phenomena attending the menopause for a long time. Again, in certain chronic cases, pain and discomfort persist for a long time, so that the operator must be careful not to promise certain and speedy relief. As the writer puts it : "Miracles are not to be expected here ; neither is it fair to promise them. The same careful consideration of all the probabilities of the case should be here weighed ; the same honest expectation of life and health afforded—no more, no less."

---

Dr. Lee, (*Philadelphia Polyclinic*, July, 1893,) in a lecture upon Massage in the Treatment of Sprains and other Injuries about

Joints, discusses this important class of injuries in a way that cannot fail to be of interest and profit to the general practitioner as well as the surgeon. Remarking that both Nelaton and Gross believed that a large majority of cases requiring amputation result from sprain, Dr. Lee adds that, with equal truth, a large proportion of acquired distortions can be ascribed to a similar cause. The nature of a sprain and the conditions following are then clearly given, and from these are deduced the indications for treatment. These are (1) relief of pain; (2) restoration of the lymphatic, arterial, and venous capillaries to their normal condition; and (3) absorption and removal of the effused material.

Given a case seen immediately after accident, the first indication is met by placing the part in water, as hot as can be borne, and beginning massage at once. The writer gives some timely observations upon the pronunciation of the word "massage," and some of its derivatives, and describes the process in the following words:

The four principal procedures in massage, "kneading," "stroking," "friction," and "percussion," are each clearly described. The benefits of massage are to be explained by its power to restore to a normal condition the ability of the cells to maintain endosmosis and exosmosis. In conditions following sprains, we find the cells hindered in their power to absorb nutritive and excrete useless materials; and by mechanically aiding the cells in the performance of their functions, massage works benefit, and becomes, therefore, a scientific mode of treatment.

Friction and stroking, with a certain amount of kneading, are the manipulations commonly employed, and these with both hands. Adhesions begin to form very early, and these are to be broken up and exudations dissipated. All isolated indurations and nodules demand especial attention, and should be entirely removed. The writer thus describes his method of applying massage:

The treatment is properly begun at a distance from the joint, and on the proximal side, that nearest the body—and this for two reasons: First, in order to prepare the absorbents and lymphatics to receive the exudates which we are about to remove from the injured parts; and, secondly, to gradually accustom the sensitive tissues to pressure. It is possible that at the first sitting it will not be practicable to treat the injured part directly at all. It may be necessary, at first, to confine our efforts entirely to what the Germans call preliminary massage, which will prepare the way for the direct treatment.

If, for instance, we are treating a sprained ankle, we begin above the knee, and, encircling as much of the thigh as possible, with the full hands, stroke upward to the pelvis. Then we start at the middle of the thigh and knead the muscles, working slowly upward to the pelvis. Next we subject the lower half of the thigh to the same process, still working from below up. This is again followed by upward stroking, considerably firmer pressure being made than at the first time. The result of this will be that the muscular capillaries and the absorbents of this large amount of highly vascular tissue will have been stimulated to increased activity, and its capacity for receiving blood augmented. Coincident with this we may be already able to observe that the tissues about the ankle are becoming less tense and swollen.

The point is then found nearest to the seat of injury, at which pressure can be borne without serious pain. Stroking is started from this point and carried up to the knee. The upper third of the leg, including the belly of the gastrocnemius, is then kneaded, and the stroking repeated somewhat more vigorously. It may be wise to stop at this point for the day. A flannel or pure rubber bandage should then be very carefully applied, and the patient instructed to keep the foot elevated. It may be, however, that the sensitiveness will already have been so much diminished that the patient will bear gentle friction with the thumb or the fingers about the periphery of the inflamed area. If so, this may be practised with one hand while the other performs upward stroking from the same locality.

Fifteen minutes is sufficiently long for each séance, and it is often well to give two each day. By thus carefully observing and taking advantage of the diminishing area of sensitiveness, we shall soon find that pressure can be tolerated over the entire injured region. The routine will be the same each day.

About the third or fourth day, movements of the foot may be begun. At first, entirely passive, later on, acto-passive, the patient making the motion while the operator resists. The movements will consist of flexion, extension, and rotation. These will, of course, produce pain at first, but the patient must be encouraged to endure this as much as possible by holding out the hope of speedier recovery.

Douching with hot and cold water alternately is a valuable adjunct in restoring tone to the relaxed vessels.

It is not too much to say that the mode of treatment above detailed diminishes the time required as compared with that of fixation, in the ratio of days to weeks, and that the ankyloses and contractions so often following the latter are almost entirely avoided.

The statistics of the Prussian army, on the surgeons of which a practical acquaintance with massage is obligatory, are convincing on this point.

If the sprain is a severe one, it will be well to put the patient on crutches for a few days, but he should be encouraged to begin to bear a portion of his weight on the joint as early as possible. A firm, elastic bandage, evenly applied, and, later on, a woven elastic anklet, will aid materially in preventing swelling and congestion.

